# Discolouring 3D Gel Dosimeter for UV Dose Distribution Measurements

**DOI:** 10.3390/ma15072546

**Published:** 2022-03-30

**Authors:** Malwina Jaszczak, Elżbieta Sąsiadek-Andrzejczak, Marek Kozicki

**Affiliations:** Department of Mechanical Engineering, Informatics and Chemistry of Polymer Materials, Faculty of Material Technologies and Textile Design, Lodz University of Technology, Żeromskiego 116, 90-543 Lodz, Poland; elzbieta.sasiadek@p.lodz.pl (E.S.-A.); marek.kozicki@p.lodz.pl (M.K.)

**Keywords:** TBO–Pluronic F–127 dosimeter, 3D UV dosimeter, UV sensor, UV radiation, Toluidine Blue O, Pluronic F–127

## Abstract

This work reports on a new TBO–Pluronic F–127 three-dimensional (3D) gel dosimeter for UV light dose distribution measurements. The optimal gel composition was found to be 60 µM Toluidine Blue O (TBO), which acts as a UV-sensitive compound; 5% *w*/*w* hydrogen peroxide (H_2_O_2_), which is necessary for initiation of TBO photodegradation and 25% *w*/*w* poly(ethylene oxide)-*block*-poly(propylene oxide)-*block*-poly(ethylene oxide) (Pluronic F–127), which forms a physical gel matrix. The dosimeter becomes discoloured when exposed to UV radiation and a discolouration is the more intense, the higher the absorbed dose is. The samples after irradiation with UVA, UVB and UVC radiation were measured using UV-Vis spectrophotometry to obtain the basic dose–response characteristic of the dosimeter, including dose sensitivity, linear and dynamic dose range, threshold dose, stability over time and dose–response for fractioned and non-fractioned doses. Additionally, the TBO–Pluronic F–127 gel dosimeter was investigated for spatial stability and the ability to measure the dose distribution of UV radiation. The results obtained indicate that the TBO–Pluronic F–127 dosimeter is a promising UV sensor and 2D/3D UV dosimeter.

## 1. Introduction

Ultraviolet (UV) radiation has a beneficial effect on human health (vitamin D3 and endorphins synthesis); however, excessive exposure to UV rays may be associated with a number of health problems, especially damage to skin (burns, cancer, premature ageing) and eyes (cataracts, degeneration of the macula lutea) [1,2,3]. Therefore, many sensors and dosimeters of UV radiation have been elaborated. These are, in particular, one-dimensional (1D) and two-dimensional (2D) systems, such as photodiodes and actinometers [4]; liquid crystal mixtures; solutions of photoluminescent dyes [5,6]; inorganic materials [7]; biological dosimeters based on polymers containing bacterial spores [8]; vegetative bacteria [9] and DNA fragments of bacteriophages [10]; as well as chemical dosimeters, including 2D polymer films containing radiation-sensitive compounds such as tetrazolium salts [11,12], polydiacetylenes [12] or triphenylmethane dyes [13]; and systems based on the surface modification of flat textiles with radiation-sensitive compounds by textiles padding [14,15,16] or screen-printing [17,18]. However, the 1D and 2D dosimeters enable measurements in a point or a plane, respectively. Therefore, they do not allow sufficient high-resolution measurements of UV dose distribution in three dimensions (3D). For this purpose, the use of three-dimensional (3D) dosimeters is required, which so far have not been examined as widely as 1D and 2D dosimeters.

The reported 3D compositions are those composed of 2-hydroxyethyl acrylate and poly(vinyl alcohol) [19] as well as those containing a poly(ethylene oxide)-*block*-poly(propylene oxide)-*block*-poly(ethylene oxide) (Pluronic F–127) matrix and UV-radiation-sensitive compounds such as leuco malachite green (LMG) [20], tetrazolium salts (nitro blue tetrazolium chloride (NBT) and 2,3,5-triphenyltetrazolium chloride (TTC)) [18,20], as well as leuco crystal violet (LCV) [21]. The use of a Pluronic F–127 gel matrix ensures high transparency and thermal stability of dosimeters [20,22,23]. Moreover, Pluronic F–127 is a synthetic, nontoxic copolymer approved by the Food and Drug Administration (FDA, New Hampshire, Maryland, USA). The 3D dosimeters based on Pluronic F–127 are soft-tissue-mimicking due to the water content in the composition (>60%). For this reason, they may be useful as tissue-resembling systems for UV radiation dose distribution measurements related to skin damage. The 3D UV dosimeters might also be suitable for UV dose distribution characterization in photochemical research. Furthermore, they could be used as UV sensors in places of UV radiation exposure. Additionally, the characteristics of the 3D dosimeter in terms of UV radiation may supplement the results of research on 3D dosimeters for 3D ionizing radiation dose distribution measurements in radiotherapy. The study of the impact of UV radiation enables precise determination of the rules for the storage and protection of such systems against accidental irradiation (e.g., during storage, transport or measurements).

This work reports on a new 3D dosimeter based on Pluronic F–127 gel matrix for 3D UV radiation dose distribution measurements. The Toluidine Blue O (TBO) dye has been chosen as a radiation-sensitive compound in dosimeter composition. So far, TBO was pretested as a component of a 3D dosimeter with a gelatine matrix for dose distribution measurements of ionizing radiation in radiotherapy dosimetry [24]. In this study, TBO was used as an active compound in a 3D dosimeter with the Pluronic F–127 matrix for use in a 3D UV dosimetry. The dosimeter was characterized relative to its chemical composition and its dose–response for various types of UV radiation. The feature which distinguishes the TBO–Pluronic F–127 gel dosimeter from other 3D UV dosimeters [19,20,21] is that it does not become coloured, but bleaches under exposure to UV radiation [24].

## 2. Materials and Methods

### 2.1. Dosimeters Preparation

The TBO–Pluronic F–127 gel dosimeter was prepared using poly(ethylene oxide)-*block*-poly(propylene oxide)-*block*-poly(ethylene oxide) (Pluronic F−127, BioReagent, Sigma-Aldrich, Saint Louis, MO, USA) as a gel matrix and Toluidine Blue Chloride (TBO, Sigma-Aldrich, Saint Louis, MO, USA) as a radiation-sensitive compound. Additionally, hydrogen peroxide (H_2_O_2_, Chempur, Piekary Slaskie, Poland) was added to the composition to initiate a photodegradation of TBO [25]. At first, the aqueous stock solution of TBO was prepared. TBO was dissolved in water with thorough stirring for 30 min at room temperature using a magnetic stirrer. An appropriate amount of TBO stock solution and hydrogen peroxide were then added to the distilled water while stirring. Afterwards, the solution was placed in a refrigerator (4 °C) for 5 min and then mixed with a 33% *w/w* aqueous solution of Pluronic F–127 prepared 72 h earlier and kept at 4 °C. The procedure of the Pluronic F–127 solution preparation is described elsewhere [22]. Finally, the resulting solution was poured into glass cuvettes (4.5 cm^3^, 1 cm optical path) or round plastic containers (2.5 cm^3^, 33 mm inside diameter). The cuvettes and containers were closed with plastic caps. All samples were stored at room temperature for 1 h before irradiation for conversion into physical gels. The samples during and after preparation had access to air and were protected against sunlight and artificial light with aluminium foil against accidental irradiation.

The TBO–Pluronic F–127 gel dosimeter consists of over 99% non-toxic components (water, H_2_O_2_ and Pluronic F–127). The only toxic component of the dosimeter is TBO, which is used in the composition in a very small amount (0.0018%). TBO is a phenothiazine type dye that has a mutagenic effect, is toxic to the organism and cells and has a toxic interaction with RNA [26]. However, the TBO-Pluronic F–127 dosimeters developed for 3D UV dose distribution measurements are placed in the glass or plastic containers. Thus, they do not come into contact with the skin and are not harmful to humans.

### 2.2. Irradiation

The radiochromic gel dosimeter samples were irradiated in the UV-curing cabinets (UVP, Upland, Canada) at three wavelengths corresponding to type A of ultraviolet radiation (UVA, 8 W, type F8T5 Blacklight, range: 315–400 nm; a peak at 369 nm, Hitachi, Tokyo, Japan), type B of ultraviolet radiation (UVB, 8 W, type G8T5E, range: 280–360 nm; a peak at 306 nm, Sankyo Denki, Tokyo, Japan) and type C of ultraviolet radiation (UVC, 8 W, type G8T5, 253.7 nm, Sankyo Denki, Tokyo, Japan). Each cabinet was equipped with five UV lamps. A given UV dose (J/cm^2^) was delivered automatically using a built-in detector and the control system of the device. The samples in the glass cuvettes were irradiated fractionally (the gap between dose fractions was equal to approximately 3 min) with UVA, UVB and UVC in the dose range of 0–3 J/cm^2^, whereas the samples in round plastic containers were irradiated non-fractionally with 0.5 J/cm^2^ of UVA, UVB and UVC radiation. Additionally, for spatial stability and dose distribution measurements, the samples in the round plastic containers were partially covered by aluminium foil and irradiated non-fractionally with 1 J/cm^2^ of UVB radiation (as a result, the covered part of the sample was bleached, and another part remained blue-coloured). Both the samples in cuvettes and plastic containers were irradiated without covering them with caps.

### 2.3. Spectrophotometric Measurements

A UV–Vis spectrophotometer (190–1100 nm, Jasco V-530, Tokyo, Japan) was used to assess the changes in absorbance of the non-irradiated and irradiated gel dosimeter samples placed in the glass cuvettes over the wavelength region of 450–750 nm. The measurements were performed at room temperature without external access to sunlight and artificial light. The irradiated samples were measured immediately after irradiation. A background absorbance spectrum of the cuvette filled with distilled water was subtracted from each gel absorbance spectra.

### 2.4. Spatial Stability of Dose Distribution

The spatial stability of UV dose distribution for the TBO–Pluronic F–127 gel dosimeter was assessed visually over time after partial irradiation of a gel sample in a round container placed on graph paper. For this purpose, the gel dosimeter was irradiated with 1 J/cm^2^ UVB radiation to half of the container and was observed for 8 days after irradiation. However, to present a possibility of recording 2D/3D dose distribution by the dosimeter, the gel in the round container was partially irradiated with 1 J/cm^2^ UVB radiation; the irradiation zone was an oval shape. Then, the sample was photographed, and the picture obtained was split into RGB colour scale channels (ImageJ, NIH, Bethesda, Maryland, USA). Afterwards, the profiles of the partially irradiated TBO–Pluronic F–127 sample were designated for grey and RGB channels. In both cases, the gels were prepared as described in Section 2.1. and irradiated with UVB radiation 24 h after preparation. The non-irradiated areas were protected with aluminium foil during irradiation in the UVB cabinet.

## 3. Results and Discussion

### 3.1. Selection of the Dosimeter’s Chemical Composition

**Figure 1:**
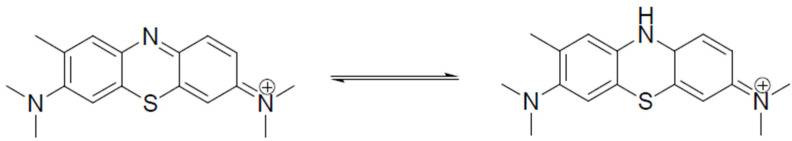
Discolouration of the Toluidine Blue dye (based on Figure 1 from [32]).

The first part of the study was to select the best-performing chemical composition of TBO–Pluronic F–127 dosimeters for further research. For this purpose, five compositions containing the same concentration of Pluronic F–127 (25% *w/w*) and different concentrations of Toluidine Blue O dye and hydrogen peroxide were irradiated with 0.5 J/cm^2^ of UVA, UVB and UVC radiation (during irradiation, the samples were not covered with caps). TBO turns the hydrogel blue in colour and acts as a radiation-sensitive compound. This dye degrades during exposure to UV irradiation, which is visible as hydrogel bleaching. The presence of hydrogen peroxide in the composition is necessary to initiate a photodegradation of the TBO process. Different reactions can take place, resulting in products interacting with TBO that lead to the discolouration of this dye. Some of them are presented below (1–6) based on the mechanisms described elsewhere [25,27,28,29,30,31]. For example, the hydroxyl radicals generated upon photolysis of hydrogen peroxide and the superoxide anion radicals formed by the reaction of hydroxyl radicals with hydrogen peroxide can react with TBO molecules, causing their discolouration due to the change in the structure of the TBO dye into the colourless leuco form (Figure 1).

(1) H_2_O_2_ $\overset{h\nu }{\to }$ $2{\mathrm{OH}}^{\cdot }$
(2) ${\mathrm{OH}}^{\cdot }$ + H_2_O_2_ → ${\mathrm{HO}}_{2}{}^{\cdot }$/$O_{2}{}^{-\cdot }$ + H_2_O
(3) TBO $\overset{h\nu }{\to }$ TBO*
(4) TBO* + H_2_O_2_ → ${\mathrm{OH}}^{\cdot }$ + ${\mathrm{OH}}^{-}$ + TBO
(5) TBO + ${\mathrm{OH}}^{\cdot }$ → Leuco form of TBO
(6) TBO + $O_{2}{}^{-\cdot }$ → Leuco form of TBO

The effects of TBO–Pluronic F–127 hydrogels irradiation with various types of UV radiation are presented in Table 1. It was found that TBO–Pluronic F–127 hydrogels react most strongly to UVB radiation (the discolouration is the most intense). Moreover, it was confirmed that the presence of H_2_O_2_ in the gel composition is necessary for the reaction to occur—composition 5 in Table 1, without H_2_O_2_, did not bleach under the exposure of UV radiation in the dose range examined.

**Figure 2:**
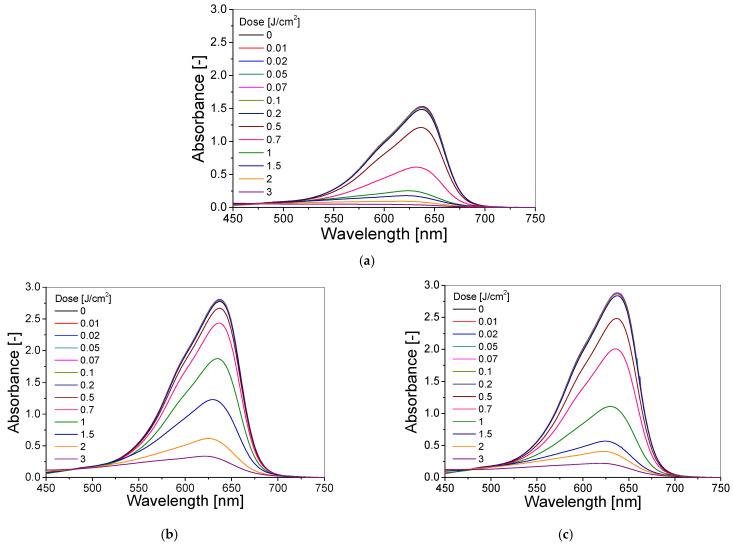

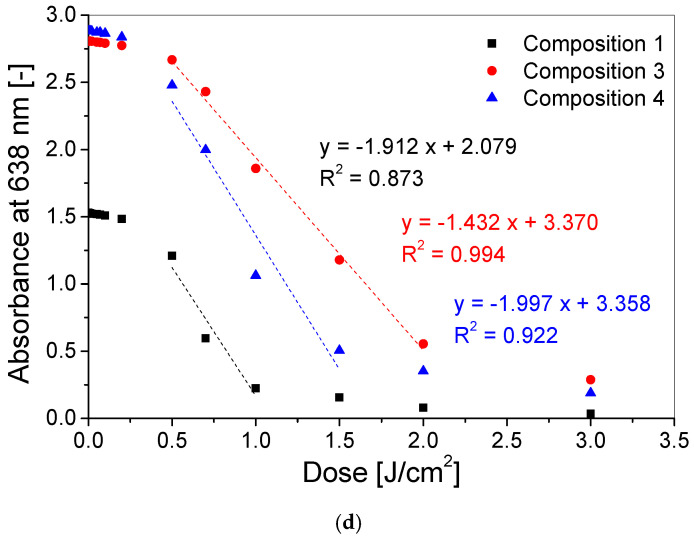
The absorbance spectra of TBO–Pluronic F–127 dosimeter containing different concentrations of TBO and H_2_O_2_: composition 1 (**a**), 3 (**b**) and 4 (**c**) (see Table 1) irradiated with UVB radiation in the dose range of 0–3 J/cm^2^ and the dose–response of these compositions at a specific wavelength (**d**).

Further in this study, three dosimeter compositions (1, 3 and 4; see Table 1) were selected to assess the effect of TBO and H_2_O_2_ concentrations on the UVB dose–response of the dosimeter (Figure 2). The same threshold dose (0.01 J/cm^2^) and dynamic dose range (0.01–3 J/cm^2^) were obtained for all compositions, and the dose sensitivities (slopes of the linear regressions of Absorbance = f(dose)) were equal to −1.912 ± 0.498 cm^2^/J, −1.432 ± 0.057 cm^2^/J and −1.997 ± 0.331 cm^2^/J for compositions 1, 3 and 4, respectively (Table 2). Composition 3 was characterized by a wider linear dose range (0.5–2 J/cm^2^) compared to composition 1 (0.5–1 J/cm^2^) and composition 4 (0.5–1.5 J/cm^2^) (Table 2). After analysing the results, composition 3 was selected for further research because of the higher absorbance of the non-irradiated sample compared to composition 1, and wider linear dose range and a correlation coefficient (R^2^) closer to 1 compared to composition 4.

### 3.2. Dose–Response of Dosimeter

**Figure 3:**
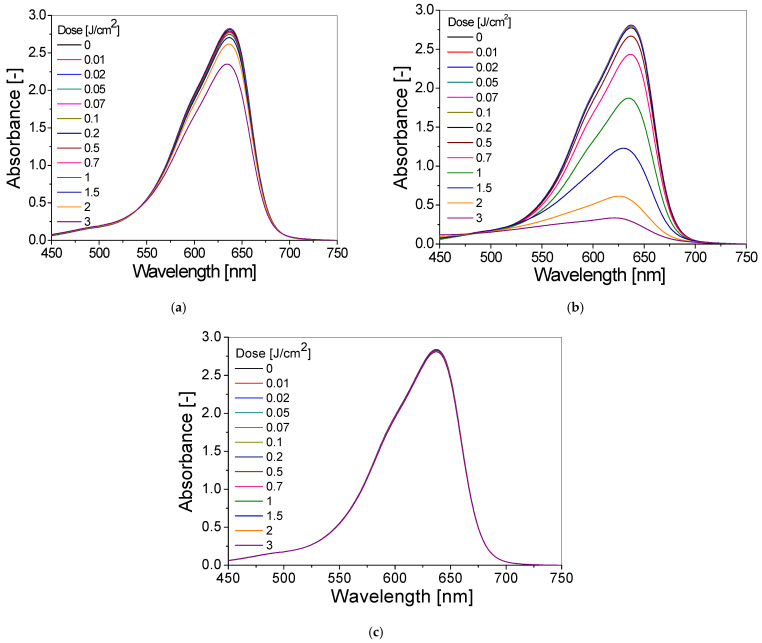
The absorbance spectra of optimal TBO–Pluronic F–127 dosimeter (60 µM TBO, 5% H_2_O_2_, 25% Pluronic F–127) irradiated with UVA (**a**), UVB (**b**) and UVC (**c**) radiation in the dose range of 0–3 J/cm^2^.

The chosen TBO–Pluronic F–127 gel dosimeter (composition 3; see Table 1) has been characterized more widely. The absorption spectra of this gel irradiated with UVA, UVB and UVC radiation are presented in Figure 3. Exposure of TBO–Pluronic F–127 gel dosimeter to UV radiation causes a discolouration of the blue gel, and bleaching is greater the higher the absorbed dose. It is expressed as a decrease in absorbance value with an increasing absorbed dose in the wavelength range of 525–700 nm with a maximum at 638 nm (Figure 3). The most intense colour fading of the gel samples occurs during irradiation with UVB (Figure 3b) and the weakest during exposure to UVC light (Figure 3c). For instance, the absorbance at 638 nm of the sample irradiated with 1 J/cm^2^ of UVB radiation is 32% and 34% lower than the absorbance of samples irradiated with the same dose of UVA and UVC radiation, respectively (Figure 3a–c).

**Figure 4:**
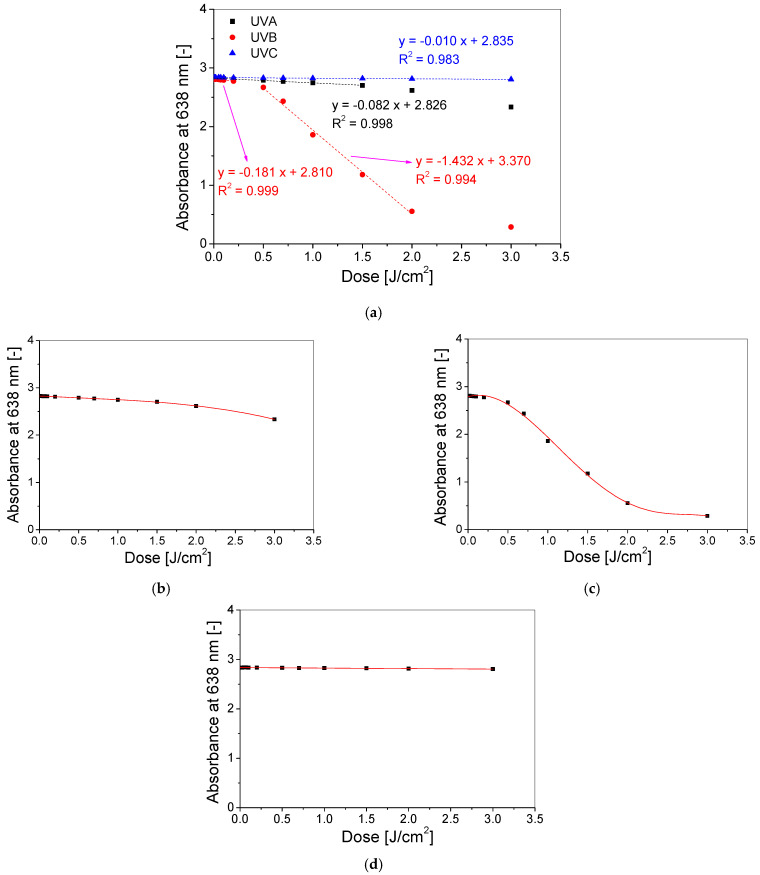
The dose–responses of TBO–Pluronic F–127 gels (60 µM TBO, 5% H_2_O_2_, 25% Pluronic F–127) with the determined linear dose ranges for samples irradiated with UVA, UVB and UVC radiation (**a**) and with fitted curves for the dynamic dose ranges of samples irradiated with UVA (polynomial curve, (**b**)), UVB (polynomial curve, (**c**)) and UVC (linear curve, (**d**)) radiation.

On the basis of the absorbance spectra (Figure 3), the dose–response characteristic was prepared, expressed as a relation between the absorbance at a specific wavelength (at which the absorption spectrum of the gel was maximal) and the absorbed dose (Figure 4a). Following this, the basic parameters for the dosimeter exposed to various types of UV radiation were extracted and presented in Table 3.

The TBO–Pluronic F–127 hydrogel reacts to low doses of all UV radiation types, from 0.05 J/cm^2^ for UVA and UVB and from 0.1 J/cm^2^ for UVC radiation. The dynamic dose range is observed from the threshold dose to the maximum dose examined (3 J/cm^2^) for each UV subrange. The dose–response of TBO–Pluronic F–127 is described by a linear equation for UVC and by a polynomial equation for UVA and UVB radiation (Figure 4, Table 3). The dosimeter responds to irradiation linearly up to 1.5 J/cm^2^ for UVA and to 3 J/cm^2^ for UVC radiation. In the case of UVB, two linear dose ranges can be indicated, and these are 0.01–0.2 J/cm^2^ and 0.5–2 J/cm^2^. The dose sensitivity of the dosimeter irradiated with UVB derived from the lower linear dose range is 2.2 and 17.6 times higher than for UVA and UVC, respectively, and derived from the higher linear dose range is 17.6 times and 140 times greater than for UVA and UVC, respectively. TBO–Pluronic F–127 is more sensitive to UVB radiation (1.43 ± 0.06 cm^2^/J) than other 3D UV dosimeters, such as LCV–Pluronic F–127 (−1.34 ± 0.15 cm^2^/J [21]) and NBT–Pluronic F–127 (0.069 ± 0.001 cm^2^/J [18]), less sensitive to UVB than LMG-Pluronic (42.85 ± 1.53 cm^2^/J [20]) and similarly sensitive to UVB as TTC–Pluronic F–127 (1.48 ± 0.06 cm^2^/J [20]), which is the most sensitive to UVA radiation.

**Figure 5:**
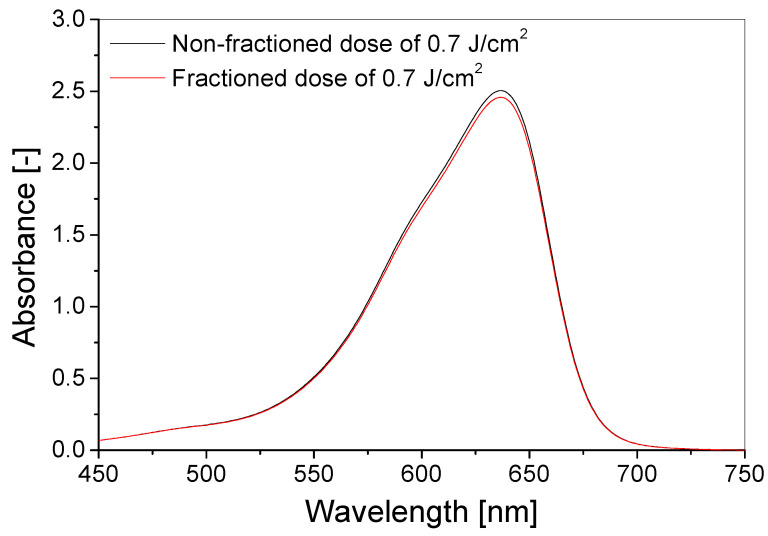
A comparison of TBO–Pluronic F–127 gels (60 µM TBO, 5% H_2_O_2_, 25% Pluronic F–127) irradiated with fractioned and non-fractioned doses equal to 0.7 J/cm^2^ of UVB radiation.

The dose–response of the TBO–Pluronic F–127 dosimeter was also assessed for the dose of 0.7 J/cm^2^ UVB delivered fractionally and non-fractionally (Figure 5). The fractioned dose was given in seven fractions of 0.1 J/cm^2^, and the gap between fractions amounted to approximately 3 min. The gels were measured immediately after irradiation. Judging by the results obtained, dosimeters react similarly to different irradiation methods applied. The absorbance at 638 nm was 1.9% higher for the dosimeter irradiated with a non-fractioned dose than with a fractioned dose of UVB.

### 3.3. Stability over Time

**Figure 6:**
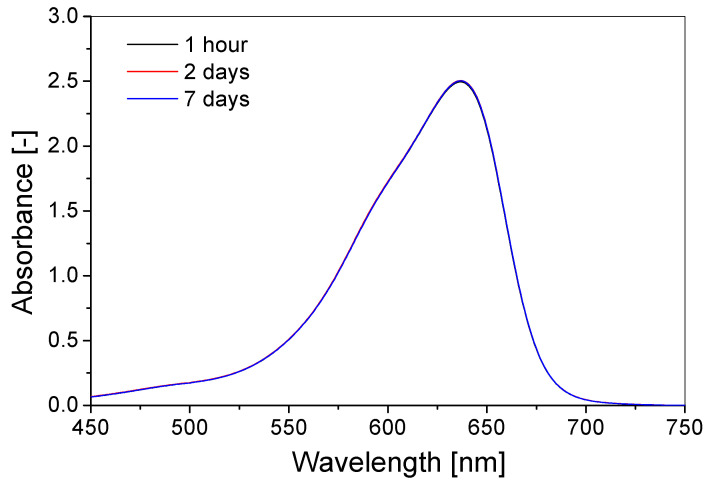
The stability of the gel-hydrogel solution (60 µM TBO, 5% H_2_O_2_, 25% Pluronic F–127) kept in a fridge. The samples were irradiated with 0.7 J/cm2 of UVB radiation and measured 1, 48 and 168 h after gel-hydrogel preparation.

The stability over time of the TBO–Pluronic F–127 gel dosimeter was examined based on the spectrophotometric measurements. In the first step, the stability of the gel dosimeter solution was assessed after preparation. The solution was kept in a refrigerator (4 °C) throughout the 7 day measurement period. It was poured into a cuvette 1 h before measurement and stored at room temperature to form a physical gel. The overlapping dosimeter spectra (Figure 6) are evidence of the stability of the gel solution for at least 7 days after preparation when kept in the refrigerator.

**Figure 7:**
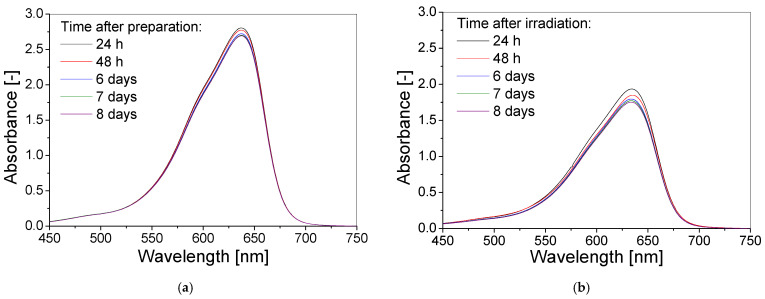

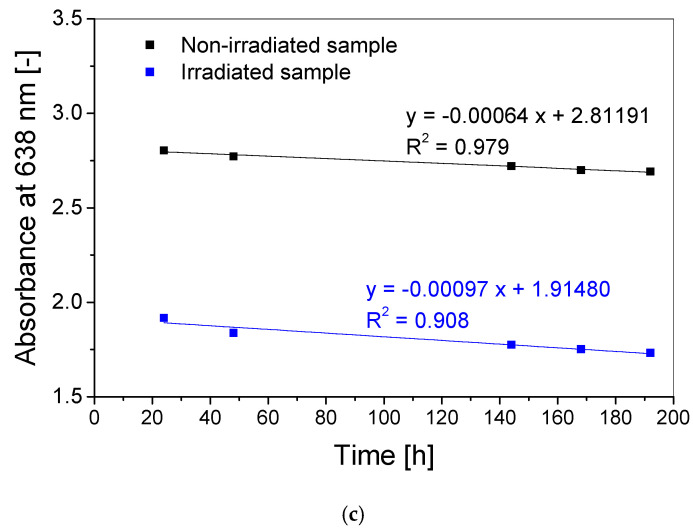
The stability over time of TBO–Pluronic F–127 gel dosimeter (60 µM TBO, 5% H_2_O_2_, 25% Pluronic F–127) non-irradiated and irradiated with 1 J/cm^2^ of UVB radiation expressed as absorbance spectra (**a**,**b**), for non-irradiated and irradiated gels, respectively) and as absorbance at maximum wavelength versus time after preparation (for non-irradiated samples) or irradiation (for irradiated samples) (**c**).

Further in this research, the stability over time of storage of the non-irradiated and irradiated TBO–Pluronic F–127 gel samples were examined. During the experiment, the samples were stored at room temperature. The results obtained show that the non-irradiated TBO–Pluronic F–127 gel dosimeter was more stable than the irradiated one (Figure 7). The absorbance at 638 nm of the TBO–Pluronic F–127 gel dosimeter decreases by 4% and 9.7% for non-irradiated and irradiated gels, respectively, within an 8 day period. In both cases, the decrease in absorbance is linear in the studied time range (Figure 7c).

### 3.4. Spatial Stability of Dose Distribution

**Figure 8:**
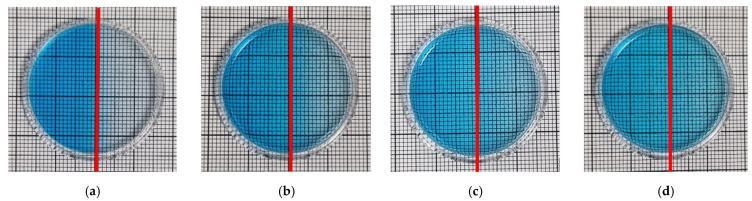
Photographs of TBO–Pluronic F–127 over time after irradiation with 1 J/cm^2^ UVB to half of the container: immediately (**a**), 1 day (**b**), 5 days (**c**) and 8 days (**d**) after irradiation. Red lines denote the position of a border between irradiated and non-irradiated parts of the dosimeter.

The possibility of using the TBO–Pluronic F–127 gel dosimeter for 2D/3D dose distribution measurements of UV radiation was investigated. In this respect, an experiment was conducted in which half of the dosimeter was irradiated with UVB radiation, and the other half was not irradiated. Photographs of the dosimeter sample over time after irradiation are shown in Figure 8, where the red line separates the irradiated part from the non-irradiated part. The obtained results indicate that diffusion from the irradiated to the non-irradiated part takes place soon after irradiation, and it occurs in the entire volume of the sample 8 days after irradiation. For this reason, the dosimeter allows reliable measurements to be made up to 1 h after irradiation. Therefore, in further work, the chemical composition of the dosimeter should be modified to obtain greater stability, which is required for a reliable evaluation of the 2D/3D dose distribution.

**Figure 9:**
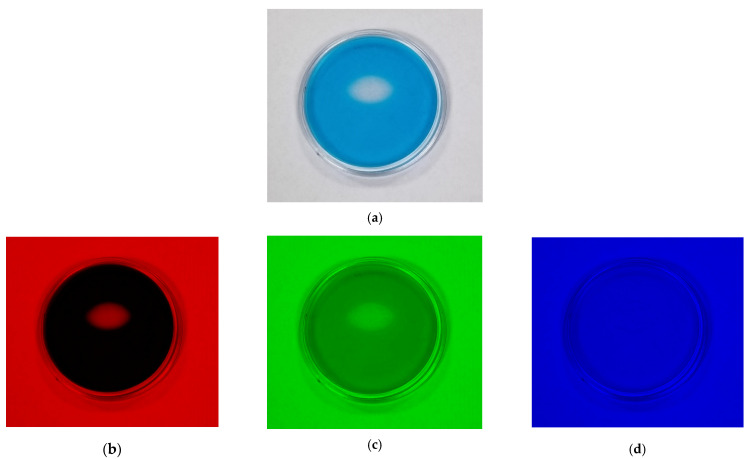
The TBO–Pluronic F–127 gel sample irradiated in the designated ellipse-shaped part (**a**) and decomposed into RGB channels: red (**b**), green (**c**) and blue (**d**).

Further in this research, the possibility of measuring the UV radiation dose distribution with the TBO–Pluronic dosimeter was checked. For this purpose, the gel placed in a round container was irradiated so that only a defined area absorbed the dose of 1 J/cm^2^ of UVB radiation 24 h after preparation. The irradiated area was elliptical in shape with a length of approximately 10 mm and a width of approximately 6 mm. The raw sample image (Figure 9a) was decomposed into RGB channels (Figure 9b–d) using the ImageJ software package. The red channel turned out to be the most sensitive to changes occurring after the irradiation of the sample.

**Figure 10:**
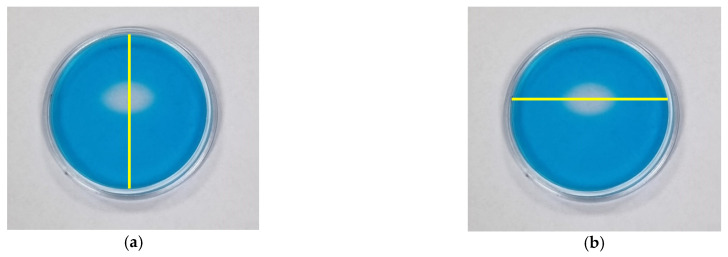

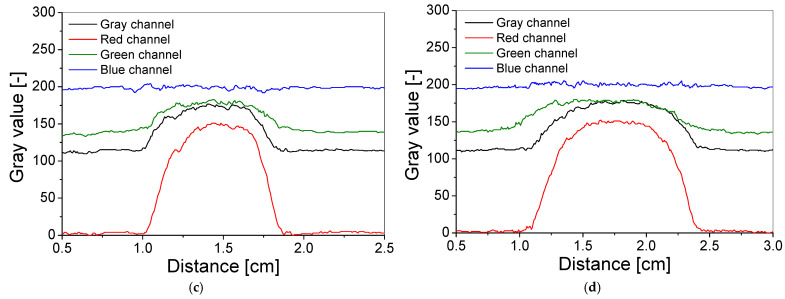
The photographs of partially irradiated TBO–Pluronic F–127 sample (**a**,**b**) and the profiles across (**c**) and along (**d**) the sample for a raw image (grey channel) and all RGB channels (red, green and blue) (**c**,**d**). The yellow lines in the photographs (**a**,**b**) indicate the position of profiles (**c**,**d**).

The profiles across and along the sample were plotted, as shown in Figure 10c,d. The increase in intensity for the raw sample (grey channel) corresponds to the irradiated part of the sample, which became discoloured. The greatest increase in colour intensity was observed for the red channel, which best illustrates the changes occurring in the sample after irradiation. The changes are not visible for the blue channel due to the analogous colour of the sample. The obtained results clearly indicate the possibility of using TBO–Pluronic F–127 to record the 2D dose distribution. It is also possible to extend the recording to 3D dose distribution measurements using a 3D optical scanning technique, which is planned for subsequent studies.

## 4. Conclusions

In this work, the TBO–Pluronic F–127 gel dosimeter was elaborated, and its dose–response for UVA, UVB and UVC radiation was assessed. For the first time, a 3D UV gel dosimeter was developed, which does not become coloured but bleaches under exposure to UV radiation. The discolouration of the dosimeters is more intense with a higher absorbed dose. The TBO–Pluronic F–127 dosimeter was shown to respond with the highest sensitivity to UVB radiation. The selected composition with the best parameters comprises 60 µM Toluidine Blue O, 5% *w/w* hydrogen peroxide, 25% Pluronic F–127 and water. The dose sensitivity of this composition equals −1.432 ± 0.057 cm^2^/J, the intercept is 3.3703 and the threshold dose, linear and dynamic dose range are 0.01 J/cm^2^, 0.5–2 J/cm^2^ and 0.01–3 J/cm^2^, respectively (for UVB irradiation). The TBO–Pluronic gel is more stable before irradiation than after irradiation. Additionally, it loses a dose distribution soon after irradiation. For this reason, further work is needed regarding the chemical composition of this dosimeter to increase its temporal and spatial stability. However, in this work, the great potential of the TBO–Pluronic F–127 dosimeter as a new sensor or system for 2D/3D high-resolution measurements of UV dose distribution was demonstrated.

## Figures and Tables

**Table 1:** Effect of TBO and H_2_O_2_ concentrations in TBO–Pluronic F–127 hydrogels on their UV dose–response.

Chemical Composition			Non-Irradiated Samples	Samples Irradiated with 0.5 J/cm^2^ of UV Radiation		
No.	TBO (µM)	H_2_O_2_ (% *w/w*)		UVA	UVB	UVC
1	30	5	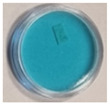	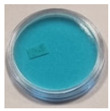	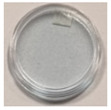	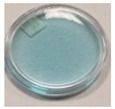
2	30	10	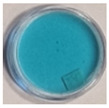	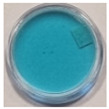	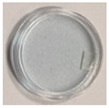	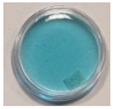
3	60	5	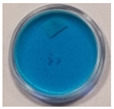	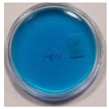	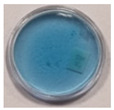	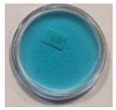
4	60	10	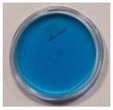	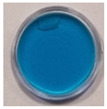	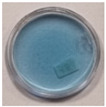	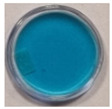
5	60	-	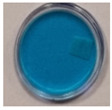	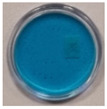	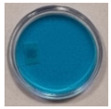	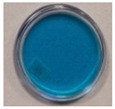

**Table 2:** Basic parameters of TBO–Pluronic F–127 dosimeters irradiated with UVB radiation.

Composition (see Table 1)	Dose Sensitivity (cm^2^/J)	Intercept (–)	Threshold Dose (J/cm^2^)	Linear Dose Range (J/cm^2^)	Dynamic Dose Range (J/cm^2^)	R^2^
1	−1.9123 ± 0.4982	2.0793 ± 0.3794	0.01	0.5–1	0.01–3	0.8729
3	−1.4315 ± 0.0573	3.3703 ± 0.0724	0.01	0.5–2	0.01–3	0.9936
4	−1.9965 ± 0.3310	3.3580 ± 0.3306	0.01	0.5–1.5	0.01–3	0.9218

**Table 3:** Basic parameters of TBO–Pluronic F–127 dosimeter (60 µM TBO, 5% H_2_O_2_, 25% Pluronic F–127) irradiated with UVA, UVB and UVC radiation, derived from the dose–responses (Figure 4). The linear equation is for the linear (for samples irradiated with UVA, UVB, UVC) and dynamic (for samples irradiated with UVC) dose range, whereas the polynomial equation is for the dynamic dose range of samples irradiated with UVA and UVB.

Type of UV Radiation	Threshold Dose (J/cm^2^)	Linear Dose Range (J/cm^2^)	Dynamic Dose Range (J/cm^2^)	Linear Equation A = a × D +A_0_ Where D Is Dose			Polynomial Equation UVA: A = A_0_ + aD + bD^2^ + cD^3^ UVB: A = A_0_ + aD + bD^2^ + cD^3^ + dD^4^ +eD^5^ where D is Dose						
				a (cm^2^/J)	A_0_ (-)	R^2^	a	b	c	d	e	A_0_	R^2^
UVA	0.05	0–1.5	0.05–3	−0.0815 ± 0.0012	2.8264 ± 0.0007	0.9978	−0.0882 ± 0.0061	0.0223 ± 0.0058	−0.0165 ± 0.0013	-	-	2.8256 ± 0.0012	0.9997
UVB	0.05	0.01–0.2	0.05–3	−0.1808 ± 0.0019	2.8099 ± 0.0002	0.9995	0.4732 ± 0.4103	−1.8483 ± 1.2114	0.4651 ± 1.2956	0.0906 ± 0.5654	−0.0295 ± 0.0845	2.7868 ± 0.0256	0.9976
		0.5–2		−1.4315 ± 0.0573	3.3703 ± 0.0724	0.9936							
UVC	0.1	0–3	0.1–3	−0.0103 ± 0.0004	2.8355 ± 0.0004	0.9829	-						

## Data Availability

The data supporting reported results are not stored in any publicly archived datasets. The readers can contact the corresponding author for any further clarification of the results obtained.
